# bcl-2 expression is not associated with survival in metastatic cutaneous melanoma: A historical cohort study

**DOI:** 10.1186/1477-7819-6-65

**Published:** 2008-06-20

**Authors:** Marília B Espíndola, Oly C Corleta

**Affiliations:** 1 Graduate Program in Medicine: Surgery, Universidade Federal do Rio Grande do Sul, Porto Alegre, RS, Brazil; 2 Rua Ramiro Barcelos 2300, Porto Alegre, RS, Brazil

## Abstract

**Background:**

Programmed cell death (apoptosis) has been implicated in tumor development and may affect the metastatic potential of tumor cells. The role of bcl-2, a proto-oncogene that inhibits apoptosis, has been studied in several malignancies, including cutaneous melanoma (CM). The purpose of this study was to evaluate the immunohistochemical expression of bcl-2 in 35 regional lymph node, 28 subcutaneous and 17 visceral CM metastases, correlating the findings with patient survival.

**Methods:**

In a historical cohort study patient survival was correlated with the expression of bcl-2 in regional lymph node, subcutaneous and visceral metastases of CM. Eighty slides containing surgical specimens from 50 patients diagnosed with stage III and IV CM, 28 male (56%) and 22 female (44%), were analyzed. Mean age at diagnosis was 43 years (16–74 years; median = 42 years). Mean Breslow depth was 5.01 mm (0.4–27.5 mm). The slides were submitted to immunohistochemical reaction using anti-bcl-2 monoclonal antibody and classified according to the degree of staining (< 5%; 5 to 50%; or > 50% of tumor cells stained). The relationship between bcl-2 protein expression and survival for each type of metastasis, gender and age at initial diagnosis was analyzed.

**Results:**

Mean overall survival was 33.9 months after the diagnosis of the initial metastatic lesion (range: 0 to 131 months). Twenty-four out of 50 patients (48%) had died from CM by the end of the study period. bcl-2 expression was detected in 74.3, 85.7 and 82.4% of lymph node, subcutaneous and visceral metastases, respectively. After univariate and multivariate analyses, no correlation was found between positive bcl-2 expression and overall survival for the types of metastases evaluated.

**Conclusion:**

The immunohistochemical expression of bcl-2 in metastasis alone is not a prognostic marker for CM.

## Background

The incidence of cutaneous melanoma (CM) has increased steadily in the last few years [1]. Sun exposure and sunburn, with subsequent genetic damage caused by ultraviolet radiation [2], play a major role in this increase. The prognosis of CM is positive in its initial stages; however, the five-year survival rate is only 41% in patients with regional lymph node metastases. A maximum survival of 2 years has been reported for patients with distant metastases [3].

Despite major advances in cancer treatment, surgery is still the treatment of choice for CM, since chemotherapy and radiation therapy generally produce low response rates [4,5]. Since the ultimate goal of non-surgical treatments is to induce apoptosis in tumor cells, this physiological event has recently received much attention [6]. Defects in the regulation of apoptosis have been implicated in tumor progression, metastatic spread and resistance to chemotherapy [7,8]. In recent years, several biomolecules, including B cell lymphoma/leukemia-2 (bcl-2), have been studied in CM, in an attempt to determine which lesions are more likely to respond to non-surgical treatments [9].

The bcl-2 gene is located on chromosome segment 18q21.3, in a telomere-centromere orientation [9]. The bcl-2 protein is an integral part of the cell membrane, with a molecular weight of 26 kDa, and it is found in the cell nucleus, mitochondria and endoplasmic reticulum [10]. bcl-2 acts as an apoptosis inhibitor, without any influence on cell proliferation [9].

The results obtained so far for the role of bcl-2 in CM are controversial. Although some authors have described an increase in bcl-2 during the progression of normal melanocytes to melanomas, others have observed the opposite [11-15]. Grover *et al. *[11] have reported a lower survival rate in CM patients with regional lymph node metastasis and positive bcl-2 expression.

The purpose of the present study was to evaluate the relationship between the immunohistochemical expression of bcl-2 and survival in patients with regional lymph node, subcutaneous and visceral metastases of CM.

## Patients and methods

In this historical cohort study, the survival of patients with CM represents the outcome, and the expression of bcl-2 in regional lymph node, subcutaneous and visceral metastases of CM is the variable of interest.

Eighty slides containing surgical specimens from 50 patients treated at three institutions between 1990 and 2007, 28 male (56%) and 22 female (44%), were analyzed. All patients had been diagnosed with stage III (regional metastases) and stage IV (distant metastases) CM [16]. In all cases, initial resection was performed at one of the participating hospitals. Exclusion criteria were previous diagnosis of other types of cancer, simultaneous diagnosis of secondary neoplasm, previous radiation therapy, chemotherapy or resection of metastases prior to diagnosis, initial surgery for metastasis at a different institution, incomplete resection of metastases, or death due to causes other than CM. The study was approved by the Hospital de Clínica de Porto Alegre Research Ethics Committee (IRB equivalent).

Table 1 shows the distribution of patients in terms of primary diagnosis and location of tumor and first metastasis. Mean age at initial diagnosis was 43 years, ranging from 16 to 74 years, with a median of 42 years. Mean Breslow depth was 5.01 mm, ranging from 0.4 to 27.5 mm. Clark's level ranged from II to V, of which level IV was the most prevalent (22 cases – 44%).

**Table 1:** Distribution of 50 patients with cutaneous melanoma in terms of primary diagnosis and location of tumor and first metastasis

Primary tumor diagnosis	Number
Superficial spreading CM	21
Nodular CM	15
Amelanotic	5
Acral	2
Unknown	7

Tumor location	

Trunk	30
Lower limbs	13
Upper limbs	4
Head and neck	3

Site of first metastasis	

Lymph node	35
Subcutaneous	28
Visceral	17
Uterine adnexa	1
Small intestine	4
Adrenal gland	2
Omentum	1
Colon	1
Lung	3
Liver	2
Testis	1
Breast	2

If more than one metastasis was present in the same site, only the first metastasis at each site was evaluated for bcl-2 protein expression. Thirty patients did not receive any treatments other than surgery. Of the remaining 20 patients, nine received chemotherapy, with regimens that included the following drugs: dacarbazine (DTIC), carmustine (BCNU), verapamil, cisplatin and tamoxifen, in varying combinations and for one to five treatment cycles. One patient received hyperthermic isolated lower limb perfusion with melphalan; complete response was observed immediately after treatment, but a relapse occurred later. Five patients were treated with interferon for 1 to 5 months. Five patients received radiation therapy, of whom four were treated for central nervous system (CNS) metastases and one for disease in the axilla, all with palliative intent.

### Immunohistochemistry

Sections of paraffin-embedded metastasis specimens were initially stained with hematoxylin-eosin for evaluation of tumor representation. The chosen blocks were sliced into 4-μm sections and stored in an incubator at 56°C for 24 h. After deparaffinization and hydration by immersion in xylol and decreasing concentrations of ethanol (100 to 20%) in room temperature, antigenic recovery was carried out using the microwave irradiation method. After that, the specimens were rinsed in tap water and distilled water and immersed in PBS buffer for 5 min. To block tissue enzymes that could interfere with the reaction, the endogenous peroxidase method was employed. To block unspecific reactions that could yield false-positive results, powder milk was used, with rehydration in 5% PBS buffer for 40 min, washing in tap water and distilled water and immersion in PBS for 5 min. The specific antibody reaction was carried out with bcl-2 antibody (IgG 1, kappa, 280 mg/L – Monoclonal Mouse Anti-Human bcl-2 Oncoprotein Clone 124 Code no. M0887 Lot 018; Dako Corporation, Carpinteria, CA, USA) diluted in PBS buffer (1:500). The sections were then stored in a dark chamber for 1 hour at 37°C or left at 4°C in a refrigerator overnight. The slides were individually washed three times for 5 min in PBS buffer. Secondary antibody (DAKO/LSAB – Dako Liquid DAB Large Volume Substrate-Chromogen System Code N°: K3466; Dako Corporation, Carpinteria, CA, USA) was applied for 30 minutes, followed by three 5-min baths with PBS buffer. The DAB kit was used for development. Counterstaining was carried out with Harris hematoxylin for 20 s after serial rinsing with tap water, 2% ammonia solution, ethanol and xylol.

The slides were evaluated by two independent pathologists and classified according to staining intensity (% stained cells), as follows:

- 0 (negative): less than 5% of tumor cells stained with bcl-2;

- I (weakly positive): 5 to 50% of tumor cells stained with bcl-2;

- II (strongly positive): over 50% of tumor cells stained with bcl-2.

Since statistical analysis did not reveal differences between groups I and II, they were considered as one group (bcl-2-positive) for the present analysis. Results are presented as arithmetic means, standard deviation, medians, and percentage rates. The chi-square test, Fisher's exact test, log-rank test, and Kaplan Meier method were used to evaluate the relationship between bcl-2 protein expression and survival for the three types of metastases (regional lymph node, subcutaneous and visceral metastases), gender and age at initial diagnosis, with 95% confidence intervals. Univariate and multivariate Cox regression tests were performed to evaluate the interaction between gender, age at first metastasis, survival, type of metastasis, and bcl-2 expression. A P < 0.05 was considered to be statistically significant.

## Results

Mean overall survival was 33.9 months after the diagnosis of the initial metastatic lesion (range: 0 to 131 months). Twenty-four out of 50 patients (48%) had died from CM by the end of the study period. Of the 26 surviving patients, nine (36%) had imaging exams suggestive of multiple metastatic lesions or a single unresectable metastatic lesion. The mean disease-free interval (from initial diagnosis to the diagnosis of the first metastasis) was 17.6 months (0 to 83 months) (median of 11.5 months and standard deviation of 21.5 months). Five patients (10%) had visceral and subcutaneous metastases simultaneously, and one (2%) had the three types of metastasis since the start of metastatic disease.

**Figure 1:**
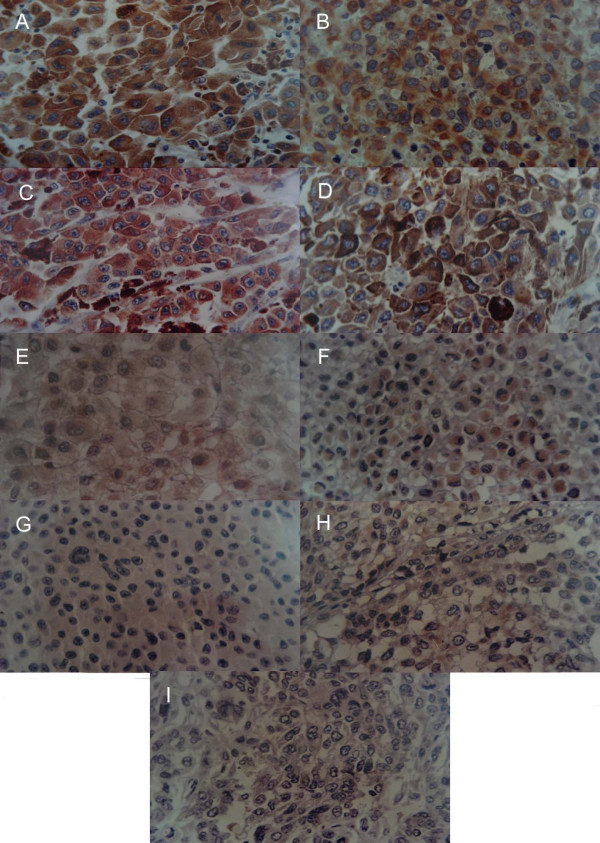
**Photomicrograh 200×.** A) Lymph node B) visceral and C) subcutaenous metastases in patients with cutaneous melanoma showing intense immunostaining (brown spots). D) Lymph node, E) subcutaneous and F) visceral mestastases in patients with cutaneous melanoma showing weak expression of bcl-2 protein. Absence of bcl-2 expression in G) lymph node H) subcutaneous melanoma and I) visceral melanoma metastases.

Results concerning the immunohistochemical expression of bcl-2 and deaths according to the three types of metastasis are shown in table 2. Figure 1 shows the different degrees of immunostaining observed in metastasis specimens, including intense (A-C), weak (D-F) and absence (G-I) of immunostaining. Fisher's exact test did not reveal an association between metastasis site and death (P = 1 for lymph node metastases, P = 0.613 for subcutaneous metastases, and P = 0.576 for visceral metastases). Table 3 shows chi-square test results for the expression of bcl-2 in the three types of metastases, revealing no difference between the sites. Fisher's exact test confirmed that the metastasis sites were similar in terms of bcl-2 protein expression, survival, and presence of unresected metastatic disease by the end of the study (Fisher's P = 1.0, P = 0.333, and P = 0.429 for lymph node, subcutaneous, and visceral metastases, respectively).

**Table 2:** Immunohistochemical expression of bcl-2 and deaths in patients with cutaneous melanoma

	Lymph node metastases		Subcutaneous metastases^a ^		Visceral metastases	
	(n = 35)		(n = 28)		(n = 17)	
			
	No.	%	No.	%	No.	%
bcl-2-negative	9	25.7	4	14.3	3	17.6
Deaths	4	11.4	3	10.7	1	5.9
bcl-2-positive						
Intense staining	17	48.6	20	71.4	11	64.7
Weak staining	9	25.7	4	14.3	3	17.6
Deaths	12	83.3	13	84.6	8	89

^a ^Stage IV [16]

**Table 3:** Chi-square test results for expression of bcl-2 in three types of metastases

	bcl-2 positive (%)	bcl-2 negative (%)	Total (%)
Lymph node	26 (74.3)	9 (25.7)	35 (100)
Subcutaneous	24 (85.7)	4 (14.3)	28 (100)
Visceral	14 (82.3)	3 (17.7)	17 (100)
Total	64 (80.0)	16 (20.0)	80 (100)

χ^2 ^= 1.34; P = 0.510.

Survival was correlated with age > 60 years at initial diagnosis (log rank = 6.17; P = 0.130) and male gender (log rank = 3.17; P = 0.0752) in patients with subcutaneous metastases. None of the other comparisons between survival, age, gender, and bcl-2 expression were statistically significant. Similar results were obtained using univariate analysis (P > 0.05) and Cox's multivariate analysis.

## Discussion

Apoptosis, or programmed cell death, has recently become the focus of great interest [7,9,17]. Discovery of the bcl-2 gene, an apoptosis inhibitor, in translocation 14;18 (q32;q21) in follicular B-cell lymphomas, followed by the identification of the BCL-2 family in the study of *Caenorhabditis elegans*, opened new perspectives for the study of tissue morphogenesis and oncogenesis [9,11,18].

Inhibition of apoptosis can occur in any phase of the cell cycle, although the exact mechanism through which bcl-2 inhibits apoptosis is not fully understood [19]. In the presence of bcl-2 overexpression, the ability of the cell to remove genetic and cell damage through apoptosis is limited. Despite the fact that bcl-2 does not act directly on cell proliferation, its overexpression enables tumors to progress to highly malignant phenotypes and to become more resistant to chemotherapy and apoptosis-inducing radiation therapy, with subsequent metastatic spread and tumor progression [8,19,20].

Several different tissues express the bcl-2 protein. In some types of neoplasms originating from these tissues (such as lymph nodes with breast cancer metastasis), a relationship between bcl-2 expression and longer disease-free survival has been observed. On the other hand, in other tumor types, there is an inverse relationship between positive bcl-2 expression and prognosis, such as in prostate cancer, ovarian cancer, non-small cell lung cancer, follicular thyroid cancer, neuroblastoma, and breast cancer [14,15]. In the case of CM, this relationship is still controversial and undefined.

Confounding variables such as sample size, gender and age should be considered when studying prognostic factors in CM. The relatively small number of cases analyzed in our study is due mainly to the absence of appropriate protocols for the management of CM in our setting, making it more difficult to gather patient information. Moreover, it is often not possible to retrieve data from inaccurate patient charts, and pathology divisions, at least in our institutions, lack an appropriate database, which would facilitate the access to patient charts using pathology diagnosis as a search variable.

The ages of 50 and 60 years have been used as cutoff points for the evaluation of CM patients [3,21-23]. In this study, we observed that patients older than 60 years were at greater risk for subcutaneous metastases of CM, and also that age had no effect on the survival of patients with other types of metastasis. The larger tumor thickness found in patients older than 60 years at diagnosis, as well as the accumulation of genetic damage acquired throughout the years, may account for these findings [24]. Fernandez-Pol and Douglas related the presence of bcl-2, mitochondrial integrity and carcinogenesis with human aging [25]. Garbe and Blum noticed that most melanomas were diagnosed between the sixth and seventh decades of life, with only 22% of the cases diagnosed before the age of 40 [26].

We were unable to evaluate the histological characteristics of primary tumors because this information was not available for all cases. Also, we did not analyze the relationship between location of the primary lesion, survival and bcl-2. Nevertheless, the characteristics of the primary lesion lose some importance once the first metastasis is diagnosed [3,22,27-29]. The importance of data about the primary tumor is in fact controversial, with different authors reporting conflicting results about the role of pathology data and primary site location in CM prognosis [1,3,21,23,27,30,31].

In addition, the expression of bcl-2 in CM metastases was evaluated without comparison to bcl-2 expression in normal melanocytes. That comparison was not performed because this is a retrospective study, based on paraffin block specimens and on medical records. It was therefore not possible to obtain normal skin or nevus samples from the patients enrolled in the study. Nevertheless, bcl-2 expression in our study was similar to that described in previous studies employing immunohistochemical methods [14,15,32,33].

We did not observe a relationship between bcl-2 expression and survival in the three types of CM metastasis. This could be explained in three distinct ways: first, there is no correlation between survival and the immunohistochemical expression of bcl-2 in CM; second, this correlation exists, but was not demonstrated in the present study due to the small sample size and short follow-up time; and third, the interaction between the other members of the BCL-2 family may have neutralized the expression of bcl-2. The regulation of proapoptotic and antiapoptotic components in the BCL-2 family is complex. Several members of this family are still being discovered, such as Bim and GRS [34,35], and the complex interactions between BCL-2 family members are only partially understood. In the future, a deeper understanding of these interactions, and mainly of their functions (rather than only of their presence), may allow for an adequate use of BCL-2 family members as effective predictors of survival in CM.

It has been shown that the treatment of melanoma cells with oligonucleotides targeting the reduction of bcl-2 expression rendered those cells more sensitive to chemotherapy [5,36-41]. These oligonucleotides are chemically modified, single-stranded DNA that complement specific codons of the messenger-RNA of a target gene, and which are capable of inhibiting the expression of this particular gene [5]. These experiments demonstrate that bcl-2 protein plays an important, although not fully understood, role in chemoresistance in CM and several other tumors [37,38].

## Conclusion

The present results suggest that the immunohistochemical expression of bcl-2 in metastases alone is not a prognostic marker for CM. Further knowledge of the actions and relations between BCL-2 family members is necessary to define the exact role of the bcl-2 protein and of other BCL-2 family members in the pathogenesis, prognosis, and response of metastatic CM to new treatments.

## Competing interests

The authors declare that they have no competing interests.

## Authors' contributions

MBE participated in study design, was in charge of data collection and analysis and helped to draft the manuscript, OCC conceived of the study, and participated in its design and data analysis and helped to draft the manuscript. Both authors read and approved the final version of the manuscript.
